# Well-being and entrepreneurship: Using establishment size to identify treatment effects and transmission mechanisms

**DOI:** 10.1371/journal.pone.0226008

**Published:** 2020-01-15

**Authors:** Christian Bjørnskov, Nicolai J. Foss

**Affiliations:** 1 Department of Economics, Aarhus University, Aarhus, Denmark; 2 Research Institute of Industrial Economics, Sweden; 3 Department of Strategy and Innovation, Copenhagen Business School, Frederiksberg, Denmark; University of Zilina, SLOVAKIA

## Abstract

Using data from the European Value Survey, covering more than 300,000 respondents in 32 countries between 2002 and 2012, we offer new insight into the consequences for subjective well-being of self-employment. We hypothesize that the positive link between entrepreneurship and well-being is influenced by the extent to which the decision to engage in entrepreneurship reflects voluntary choice and by the ability of the entrepreneur to match entrepreneurial preferences for autonomy, task variety, and challenging tasks to task environments. While the hypotheses are confirmed by our empirical analysis, we also find—rather surprisingly—no evidence that the effects are mediated by autonomy. To handle the endogeneity and simultaneity problems that arise from the fact that the choice to become an entrepreneur is not random and which potentially threaten the validity of our findings, we rely on a novel econometric method which allows us to sidestep the selection problem and establish that the well-being increase associated with entering into entrepreneurial activity is at least approximately equivalent to a one-decile increase in household income.

## Introduction

Over the last few decades, public authorities and policy-makers across the world has increasingly emphasized the need to stimulate entrepreneurship. The latter is seen as positively contributing to dynamism, “churn,” productivity and growth [1, 2]. Researchers have pointed out that the incentives for individuals to engage in such beneficial entrepreneurship may be perverse: Individuals who are self-employed on average have a lower income than those who are in regular employment [3]. However, such arguments arguably fail to take into account that the ultimate aim of tangible economic activity is utility, or, even broader, well-being or happiness [4]. Beginning with the pioneering work of [5], a small body of research reports evidence that although entrepreneurship on average does result in lower expected tangible income, entrepreneurs also on average report higher overall well-being [6, 7, 8, 9]. However, relatively little is known about the mechanisms that drive the relation between entrepreneurship and well-being, as well as the factors that influence it, in terms of theory-based accounts of moderating and mediating variables that are supported by the available evidence.

The association between entrepreneurship and well-being may be interpreted as a consequence of procedural utility [6]. In other words, individuals value not only outcomes, but also the means with which to achieve them. Specifically, self-employment affords individuals the opportunity to assume more control over their lives than those who are employed, because self-employment means the absence of the external control implied by being a member of a hierarchical organization, and because individuals with an autonomous locus of control are better able to cope with financial stress [6, 10]. The absence of such external control harmonizes with the human desire for autonomy and the ability to exercise one’s competences and the well-being consequences of such autonomy [11, 12]. However, this is only one possible mechanism linking entrepreneurship and well-being. We argue that factors such as a preference *per se* for the tasks that entrepreneurs (typically) carry out as well as a preference for task diversity may also be relevant [13, 14]. If entrepreneurs hold such preferences, we would expect the relation between entrepreneurship and well-being to be influenced by whether entrepreneurs engage in opportunity or necessity entrepreneurship [15], to the size of the firms that entrepreneurs run and to the industry they are in, as all these factors influence the extent to which entrepreneurs may satisfy these preferences. We build up these ideas as moderating hypotheses relative to our baseline hypothesis that there is a positive relation between entrepreneurship and well-being. To test our ideas, we use data from the European Social Survey to gain new insight into the consequences for well-being of being an entrepreneur, the mechanisms that produce the link between entrepreneurship and well-being, and the factors that moderate the relation. Analysis of the data confirms our main hypotheses.

Our arguments raise the methodological challenge that entrepreneurship may attract individuals with strong preferences for autonomy, as well as for engaging in specific creative tasks, or even task diversity. Thus, conclusions about a positive relation between well-being and entrepreneurship made on the basis of correlations like those we report in this research are threatened by potential endogeneity and simultaneity problems because the choice to become an entrepreneur is not random. These features create a particular problem: when estimating the difference in well-being between entrepreneurs and other seemingly comparable individuals, the estimate includes a selection effect, which derives from personality traits and other non-observed characteristics that affect the choice to become an entrepreneur and which may directly affect subjective well-being [9]. Moreover, the estimate includes a treatment effect from the actual experience of entrepreneurial activity. In simpler terms, we do not know whether the positive association is caused by those who are already high in well-being and life-satisfaction selecting into entrepreneurship, or whether entrepreneurship actually positively influences well-being, or both, or is spurious if the choice of entering entrepreneurship simply reflects non-observed characteristics directly associated with well-being. While research on subjective well-being leans towards the view that well-being is a trait that is stable over time and strongly dependent on key personality traits [16], self-determination theory in motivational psychology [11] provides strong reasons why entrepreneurship may positively influence well-being. In this research, we are mainly interested in this treatment effect.

Given the nature of the data, we cannot conclusively address this problem by direct methods. We instead rely on a method pioneered in [17] concerning the causal interpretation of moderation effects, and already used in several studies to establish causality in methodologically challenging situations [18, 19]. This method allows us to sidestep the selection problem and establish that the well-being increase associated with entering into entrepreneurial activity is at least approximately equivalent to a one-decile increase in household income. In fact, this is our most conservative estimate of the causal impact of entrepreneurship on well-being. We also apply the method to establish differences in well-being between entrepreneurs that direct small as compared to larger companies. Although the reasons for observing a difference in subjective well-being between these two kinds of entrepreneurs are unobservable, they may stem from the difference in the entrepreneur’s task-portfolios in small as compared to larger firms. In the latter firms, managerial tasks which are less challenging and creative than more genuinely entrepreneurial tasks and more tedious and routine tasks tend to dominate, leading to a relative drop in well-being. In partial support of this argument, we also find that the firm size effect on well-being is not driven by employment in industries in which individuals tend to be self-employed out of necessity. Extending this method, we also find evidence for our hypothesis that the effects of entrepreneurship apply only to those individuals who choose self-employment as an opportunity instead of necessity, as the well-being of individuals entering self-employment from a state of unemployment is unaffected by their choice (i.e., our second moderation hypothesis).

Finally, our data indicates that the self-employment effects cannot be due to entrepreneurs having particular preferences for autonomy or that they merely experience more autonomy. While the data allow us to directly control for experienced autonomy on the job, and for stated preferences for autonomy, their inclusion in the empirical analysis does not lead to any appreciable change in the estimated effect of entrepreneurship. We also find that although entrepreneurs report stronger preferences for autonomy and more actual autonomy, the patterns in these associations do not match the patterns in the effects of entrepreneurship on well-being. This is a quite surprising finding, and one that runs counter to a considerable body of established research in the entrepreneurship literature, such as [6, 20].

In sum, we go beyond existing research on entrepreneurship and well-being [4,5,6,8] in three ways. First, we present theoretical arguments why the established association between self-employment and well-being may be moderated by firm size, industry, and the previous employment status of those who transition into entrepreneurship. Second, we include a larger sample of countries than most previous studies, adopt a novel identification strategy, and present new findings, in particular with respect to the effect of well-being from entrepreneurship being firm size and industry-dependent. Finally, our data allow us to assess whether the effects of entering entrepreneurship are mediated by experienced autonomy, for which we find no evidence. Our findings have implications for the understanding of the non-pecuniary benefits of entrepreneurship, in particular how these may depend on firm size, industry and opportunity, and therefore for the understanding of some of the potential hindrances to growing entrepreneurial ventures (i.e., as the subjective well-being of an entrepreneur declines as function of the size of the venture, her incentives to expand it may be diminished).

## Theory

### Motives for engaging in entrepreneurship

The early economics literature explains entrepreneurship in terms of the pursuit of profits or rents [21, 22, 23, 24, 25, 26]. Thus, the motives of entrepreneurs are seen as one-dimensional. This is partly because the early writers were not primarily taken up with the entrepreneur *per se* [27]; rather, their interest was in understanding the consequences of entrepreneurship, such as economic fluctuations [22], the formation of firms [23], or the role of equilibration in the market process [25]. For the purpose of such “functional” conceptions of entrepreneurship [28], simple profit-oriented motives are sufficient. However, some of the early writers on entrepreneurship, notably [22], hinted that the entrepreneur may be driven by extra-economic motives, such as empire-building and the sheer desire to prevail against opposition. Similarly, while many more contemporary economics contributions to entrepreneurship research typically begin from the simple, robust assumption that the pursuit of gain is the key motive of entrepreneurs [29, 30], many contributions adopt a broader utility maximization view in which profits are not the only source of utility [31]. Such an approach is used in [3] to explain the “puzzle” that the returns from entrepreneurship may be lower than the opportunity costs of this activity, implying a 35% median earnings differential between entrepreneurs and employed individuals. The author shows that the differential cannot be explained in terms of low‐ability individuals selecting into self‐employment, and concludes that the non-pecuniary benefits of entrepreneurship may be substantial. Such explanations may be alternatives to explanations stressing (irrational) overconfidence or otherwise distorted views of one’s self-efficacy [32] as a cause of becoming self-employed.

Indeed, much entrepreneurship research now stresses such benefits, including research on social entrepreneurship [33], which suggests that social entrepreneurs are motivated by pro-social concerns and the “warm glow” and overall satisfaction that acting on pro-social motivation may cause. In other words, management scholars in the entrepreneurship field have for a long time maintained that the decision to become an entrepreneur may very well have a huge “extra-economic” part and may indeed drive the decision. Among such extra-economic parts are preferences for entrepreneurial job attributes [34], such as autonomy (i.e., the capacity to make independent, uncoerced decisions, reflecting an internal locus of control [6, 35]), risky activities [34], and task diversity (i.e., entrepreneurs typically have to engage in a wide variety of different tasks, [13, 36]). To our knowledge, among these only autonomy has been explicitly linked to well-being [6]. Research finds that entrepreneurs also spend substantial time on specific tasks such as exchanging information, doing analytical and conceptual work and product development [37], but these findings are not linked to well-being. In turn, the sources of preferences for risky activities, task diversity, etc. are arguably personality characteristics such as high self-efficacy, which may underlie a preference for autonomy as well as for task variety, and perseverance and ability to recognize opportunities, which may underlie a preference for riskiness as part of the job [38]. When individuals with personality characteristics like high self-efficacy and perseverance are matched with entrepreneurial jobs, we would expect this to positively influence their well-being.

### Entrepreneurship and well-being

Although aspects of well-being, such as utility maximization, self-realization, job satisfaction, etc. have very often been invoked in entrepreneurship research, relatively little research directly address the entrepreneurship/well-being nexus [4, 5, 6, 8]. Based on British and US data, [5] were the first to find that individuals in self-employment report much higher levels of subjective well-being than other seemingly comparable individuals. Noting that this finding could potentially arise because the self-employed simply share other non-observed characteristics that tend to make them more satisfied, [6] show in individual-level panel data from three European countries that the self-employed tend to be more satisfied with their lives than other people. Moreover, they demonstrate that individuals when starting their own firm—that is, when they become self-employed—on average become more satisfied than they were before. Additionally, individuals who leave self-employment become less satisfied. Thus, [6] alleviate the substantial self-selection problem that early studies tend to suffer from. However, a potential limitation of their study is that it is limited to individual-level data from Germany, Switzerland, and the United Kingdom. [8, 9] use a larger survey and apply matching methods to establish that individuals who appear otherwise to be similar are more satisfied when they are self-employed. Recent research examines the entrepreneurship and well-being nexus at an aggregate level, asking whether the incidence of entrepreneurship impacts on national happiness, and whether nations with happy citizens are better for entrepreneurs to start new businesses [4]. The key finding is that opportunity-based entrepreneurship contributes to country-level happiness. Happiness and entrepreneurship correlations across European cities are explored in [39]. Finally, the literature is challenged in [40] by the finding hat Germans who switch from paid employment to self-employment experience increases in job satisfaction, but not life satisfaction. In line with previous results in [41], this suggests that entrepreneurship may lead to changes in several domains of satisfaction.

In line with the existing literature, we posit that entrepreneurship is a cause of well-being, and we expect entrepreneurs to realize higher levels of subjective well-being than employed; thus, our first baseline hypothesis is this:

Hypothesis 1: *Entrepreneurship causes subjective well-being*.

We deliberately use causal language in the wording of our first hypothesis, as our identification strategy allows for (cautious) causal claims to be made. We thus also seek to respond to the argument in [42] that the absence of causal identification is one of the key challenges in literature on well-being or happiness, but explicitly without having to make assumptions about the selection process into entrepreneurship.

### Entrepreneurship and voluntary choice

Since the 2001 Global Entrepreneurship Monitor report [15], the distinction between opportunity and necessity entrepreneurship has become prevalent in the field. The distinction reflects a difference between those that transition into entrepreneurship because they recognize a hitherto overlooked opportunity and those, typically of low ability, that are forced into entrepreneurship by adverse circumstances. Conceptually, the distinction is not unproblematic, as also necessity entrepreneurs, to the extent that they succeed in becoming self-employed, may be said to have recognized an “opportunity.” However, the core of the distinction is a difference concerning the locus of the forces that drives an individual to engage in action, that is, the extent to which choice can be said to be “voluntary” [43]. Under opportunity entrepreneurship the locus of forces that make an individual engage in entrepreneurship are to a larger degree internal rather than external. A study of the link between loci of control and creativity [44] finds a significant difference on their happiness measure between those individuals with internal locus of control versus those with external locus of control. This would suggest that individuals who engage in entrepreneurship for voluntary reasons report higher well-being than those who engage in it for less voluntary reasons. In support of this, research finds that becoming unemployed is associated with a change in the perceived locus of control, from internal to external [45]. Unemployment is a reason for engaging in necessity entrepreneurship. This reasoning motivates the following hypothesis:

Hypothesis 2: *The entrepreneurship-well-being relation is substantially stronger when entrepreneurship decision is a predominantly voluntary choice*, *reflecting that individuals engage in opportunity*, *rather than necessity entrepreneurial activity*.

## The preferences of entrepreneurs

### The role of autonomy

Individuals may transition into entrepreneurship to satisfy extra-economic preferences and increase their well-being in this manner. Much interest has centered on the entrepreneurial job characteristic of autonomy. As explained in [6]:

One can be independent, or one can be subject to decisions made by others. This paper argues that this difference, embodied in the institutional distinction between the decision-making procedures “market” and “hierarchy”, affects individual wellbeing beyond outcomes. Taking self-employment as an important case of independence, it is shown that the self-employed derive higher satisfaction from work than those employed in organizations, irrespective of income gained or hours worked. This is evidence for procedural utility: people value not only outcomes, but also the processes leading to outcomes. (p. 362)

That individuals value the “processes leading to outcomes” can be explained in terms of self-determination theory from the psychology of motivation (as recognized in [6]) which argues that people have a strong preference for being in charge of the locus of control of their own actions [11]. Extrinsic motivators, such as those employed by firm hierarchies, are in general less preferred than the intrinsic motivators associated with being in control of one’s own actions, something that self-employment may further to a larger extent. Thus, this reasoning is similar, though not necessarily identical, to our reasoning in support of hypothesis 2 above.

Since [6], a number of papers have explored the role of the entrepreneurial job characteristic of autonomy. Thus, using data from the 2006 European Social Survey, [46] finds that”net of values and personality traits, autonomy and independence are the mechanisms by which self-employment leads to higher levels of job satisfaction” (p.165). [20] build a model involving a multifactor utility formulation that formalizes the notion of an explicit, autonomy-based preference for self-employment, allowing them to formalize the trade-off between increased autonomy from being an entrepreneur with the higher income afforded by employment. However, such papers tend only to address certain aspect of the well-being/entrepreneurship nexus. Most of the existing literature, such as [6, 47], deal with either job satisfaction or satisfaction with earnings, or even consider prior dissatisfaction as a cause of entering entrepreneurship [48].

#### Preferences for entrepreneurial tasks and task variety

Individuals may value autonomy rather than its opposite not just for “procedural” reasons [6], but also because autonomy enables individuals to engage with tasks that are inherently more interesting and attractive [46]. In fact, this preference rather than a preference for autonomy per se may drive the transition into entrepreneurship.

Thus, this perspective implies that the increase in well-being is caused by engaging with particular entrepreneurial tasks rather than the “procedural” aspects of being autonomous per se. Second, being “self-employed” or an “entrepreneur” may mean many things, such as running a Mom & Pop store with no growth intentions, starting a one-man firm as a way out of unemployment, or forming a new venture with a replicable business model and ambitious growth intentions. Well-being may differ across such entrepreneurs, not least between individuals becoming self-employed out of necessity and those actively choosing self-employment as a response to entrepreneurial opportunities. As argued in [8], failure to take this into account arguably represents not only a significant gap in the extant literature, but also an important methodological problem.

However, we hypothesize that the kind of activity that the entrepreneur engages in matters to subjective well-being: Some activities are likely to be inherently more satisfactory to entrepreneurs than other activities, because of personality characteristics of entrepreneurs, such as risk propensity, perseverance and the willingness to assume responsibility for assuming and concluding challenging tasks [6]. Thus, activities that are relatively open-ended, non-routine and involve choice and personal judgement are more likely to create well-being. This reasoning leads to the following hypothesis:

**Hypothesis 3:** *The entrepreneurship-well-being relation is mediated by autonomy as well as the ability of entrepreneurs to match their task environment to their specific preferences for tasks and task diversity*.

An empirically testable implication is that the relation between entrepreneurship and subjective well-being is industry dependent. However, it also emphasizes the methodological problem of separating well-being effects arising from having particular preferences from effects of making particular choices, such as becoming self-employed, as the relation hypothesized in Hypothesis 3 is more likely to hold for those predisposed for or with strong preferences for “entrepreneurial” tasks [49].

### Entrepreneurs becoming managers

The distinction between the entrepreneur and the manager was famously introduced in [22], which argued that as entrepreneurs grow their venture, they turn into managers and cease to be entrepreneurs. We hypothesize that this process has implications for the subjective well-being of entrepreneurs, for the same reason as above: Entrepreneurs in small, often upstart companies are more likely (on average) to do more “entrepreneurial things” than entrepreneurs who have already grown their venture significantly and now are engaged in “running the business.” Entrepreneurs may be thought of as individuals who assume decision-making responsibility in the context of non-routine tasks and situations that require judgement, initiative and “thinking on one’s feet” in the hope of making a profit. Entrepreneurship is often associated with decision-making in situations characterized by genuine uncertainty where no established decision rule is applicable and where judgment is required [27]

Per definition, these are non-routine situations. An empirically testable implication is that the causal relation between entrepreneurship and subjective well-being is dependent on the size of the venture. While non-routine situations arise in firms of any size, and indeed explain why management exists as a function, many of such situations arising in larger and more established firms may still be resolved by invoking precedence, principles more or less explicitly embodied in corporate culture and so on. As such, even when self-employed, individuals may perform many more standardized managerial tasks, exercise less individual judgement and creativity, and experience less autonomy and task diversity in relatively large firms. Thus, a case study of 12 entrepreneurs finds that entrepreneurs in more established firms perform more “organizational development actions” instead of product development and analytical work [36]. Individuals who originally chose to become self-employed due to having particular preferences for autonomy or specific tasks may therefore find themselves in a situation in which their actual tasks in a larger firm no longer match those preferences. Conversely, in younger and typically smaller companies, precedents and principles are less likely to exist, requiring more exercise of entrepreneurial judgment in unique situations. These differences are likely to have motivational implications: In the larger firm, the entrepreneur is more “controlled” by bureaucratic demands. In the smaller firm, the entrepreneur, in contrast, has to exercise more “autonomous” decision-making to cope with unpredictable and uncertain situations, cope less with regular management tasks, and experience larger task diversity.

To the extent that entrepreneurs prefer autonomous decision-making, they are also likely to suffer a loss in overall well-being as more controlled motivation substitutes for autonomous motivation [6]. However, in addition to the autonomy aspects of entrepreneurial choices stressed in [46], we hypothesize that at least two other aspects of self-employment and entrepreneurship may explain the well-being dividend of these choices. First, as argued above entrepreneurs may also have task-specific preferences such as innovative and creative elements of daily work that are also likely to disappear at the managerial level in larger firms. Second, they may prefer more task variety and actively embrace uncertainty as a welcome distraction from daily chores. These aspects that are not necessarily associated with increased autonomy also characterize the management and activities of many small and medium-sized or young firms. Given that individuals who actively chose to become self-employed have particular preferences for task-specificity and -variety, these factors provide an alternative theoretical explanation for the potential well-being effects of self-employment. The above reasoning motives our final hypothesis:

**Hypothesis 4**: *The entrepreneurship-well-being relation is influenced by the size of the firm that the entrepreneur directs*. *the larger the firm*, *the weaker the entrepreneurship-well-being relation*.

## Data and estimation strategy

### Data

To test our hypotheses, we employ the combined file of the first six waves of the European Social Survey [50], which includes almost 300,000 individuals in 32 European countries between 2002 and 2012. The survey aims at providing representative populations, although it deliberately oversamples immigrants. However, we take advantage of this feature by comparing immigrant and native self-employed individuals. We also separate first-generation immigrants from Western and non-Western countries, as the latter are substantially more likely to share characteristics that are specific to entrepreneurs.

### Variables

#### Dependent variable

Our first main variable is “subjective well-being”, which we measure by tapping into individual answers to the question “All things considered, how satisfied are you with your life as a whole nowadays?” The reply is given on a 0–10 scale, with 0 being the worst and 10 the best possible state. This measure has been identified in earlier research as a good proxy for the cognitive dimension of subjective well-being [16, 51]. As the more recent literature on well-being and life satisfaction, we prefer to use this single-item measure over broader measures as it is interpreted similarly across cultures and can be interpreted cardinally [52]. In addition, the single-item measure is known to capture the cognitive evaluations that individuals form about the quality of their lives. These evaluations are fundamentally different from affective and emotional reactions to events that tend to be short-lived and are only weakly correlated with cognitive evaluations [53, 54, 55]. We thus aim to avoid spurious findings arising from the use of multi-response measures that mix affective and cognitive measures. A related point is that this difference also leads us to question the value of experimental studies on subjective well-being. The literature on life satisfaction gives rise to concerns regarding experimental studies in this particular context, as a long series of studies document substantial adaptation to changing circumstances, such that satisfaction levels tend to return to their initial levels after many events. Performing experiments in our context may therefore yield misleading results, as the results may be temporary and vary quite considerably across cognitive and affective dimensions of well-being [56]. Previous work in the entrepreneurship literature has mainly focused on affective components [57].

#### Independent variable

Our second main variable is self-employment, which we get from self-reported information on employment in the European Social Survey. The ESS includes two different questions that allow us to identify individuals who are self-employed. We use the information on their employment status where respondents can report that they are self-employed. However, it is also possible to identify them from information on the organization for which they work. The information in these separate measures fortunately match up almost perfectly. In further tests, we combine the self-employment dummy with information on two factors. First, we create dummies for self-employment in three different types of establishment size in order to separate small from medium and large establishments; small are defined as establishments with fewer than 25 employees and medium are defined as having fewer than 100 employees. We divide firm size into these three broad categories as both average firm size and what is considered a “small” enterprise varies substantially across European countries [58], which makes the use of more fine-grained problematic. In addition, these firm-size categories fit the conditional findings in [59] which finds substantially smaller effects in firms larger than approximately 25 employees. Second, we interact self-employment with information in a subset of the European Social Survey on whether or not the respondent has been unemployed at any time the previous five years. We do so as an admittedly imperfect way to sort out those respondents who have entered self-employment due to necessity—that is, as a way out of unemployment—instead of engaging in opportunity entrepreneurship [10].

In further tests, we use individual answers to “How much control do you have in deciding how your own daily work is/was organised?,” and respondents agreement with with the proposition that it is “Important to make own decisions and be free” as direct measures of perceived autonomy. The former is measured on a scale from 0 (no control) to 10 (full control) while the latter is measured from 1 (very much like me) to 6 (not like me at all). We add these measures to the estimates of well-being as direct tests of the autonomy hypothesis, but also use them as dependent variables, as the hypothesis requires that entrepreneurs actually prefer and experience more autonomy than comparable individuals do in other occupations.

#### Control variables

We follow the literature on subjective well-being in our choice of control variables [16, 60]. We first add a set of trust variables, capturing social trust (trust in other people in general) and political and institutional confidence. Social trust refers to individuals’ answers to the question “in general, do you think most people can be trusted?” Political confidence is an average of confidence in the country’s parliament, politicians and political parties while institutional confidence is an average of confidence in the legal system and the police; all three trust variables are measured on a scale from 0–10. We also include how religious individuals are, as well as their self-placement on a political left-to-right scale. Both are asked on a 0–10 scale and due to particularly many missing observations with the political self-placement, we also code a dummy for missing answer.

We next add a dummy for female respondents, the respondents’ age and age squared—following the standard finding that the age relation is U-shaped—, as well as three dummies capturing respondents’ civil status: whether they are living with a partner; whether they have children at home; and whether their children have moved out [16, 61]. We also add household income (in deciles), and dummies for whether the respondent is retired or currently unemployed. We further control for respondents’ immigration status and the sector they are employed in. We add dummies for first- and second-generation immigrant, and “half immigrants”, when one of the two parents is or was a first-generation immigrant.

Finally, we are concerned with primarily identifying effects of opportunity entrepreneurship (rather than necessity entrepreneurship [8, 62]. In addition to including a dummy capturing if the respondent has entered self-employment from unemployment during the last five years, in which case the choice of self-employment may have been made out of necessity, we also code particular dummy variables for 1) self-employment in agriculture, fishery, forestry and mining, and 2) food and accommodation industries, as these industries are particularly easy to enter for necessity entrepreneurs. We therefore expect that being self-employed in these types of industries may not cause more well-being. All variables are summarized in Table 1.

**Table 1:** Descriptive statistics.

Variable	Mean	Standard deviation	Observations
Subjective well-being	6.771	2.367	289,941
Social trust	4.914	2.496	290,416
Political confidence	3.772	2.277	287,610
Institutional confidence	5.383	2.471	288,653
Religiosity	4.796	2.991	288,949
Left-right placement	5.117	2.047	291,686
No placement	.150	.357	291,686
Female	.541	.498	291,385
Age	47.581	18.556	290,258
Living with partner	.524	.499	291,686
Children at home	.386	.487	291,686
Children moved out	2.91	.454	291,686
Income decile	5.062	3.080	236,080
First generation immigrant	.089	.286	291,309
Half immigrant	.264	.631	291,686
Second generation immigrant	.102	.303	291,686
Western immigrant	1.121	.756	42,068
Retired	.255	.436	291,686
Unemployed	.066	.249	291,686
Not unemployed last 5 yrs	.053	.499	74,976
Agriculture plus	.055	.227	291,686
Food and hotel services	.050	.218	291,686
Self-employed	.100	.300	291,686
Self-employed, below 25 employees	.092	.289	291,686
Self-employed, below 100 employees	.088	.284	291,686
Autonomy, actual	6.940	2.801	203,840
Autonomy, preference	2.215	1.103	278,645

#### Estimation strategy

We estimate the association between self-employment and subjective well-being using OLS regression with country and period fixed effects. This implies that all common business cycle effects and all country-level factors that are approximately time-invariant in the 12-year span, which the data cover, are effectively controlled for. In the following, we also change between employing the full sample, excluding all immigrants, excluding first-generation immigrants, and only including immigrants who may be structurally different from native respondents. In further tests, we also distinguish between immigrants from Western and non-Western countries and thereby test for the influence of the latter type of immigrants that may be substantially more prone to engage in necessity entrepreneurship. Immigrants from non-Western countries often lack skills and are poorly integrated into European labor markets, and typically migrate from countries with high levels of necessity entrepreneurship. They are therefore substantially more likely to enter into necessity entrepreneurship than natives and more likely to do so in industries which require fewer skills and less experience. Consequently, as their motives for entering self-employment are different, we expect to see a different effect on well-being of this choice. We also deal with the same problem by excluding all respondents who are self-employed in primary industries (agriculture, fishery, forestry and mining) and in accommodation and beverage services, which are either traditionally characterized by self-employment or are comparatively easy to enter for necessity reasons.

We employ a version of the method in [17] of achieving causal inference through heterogeneity. The method involves employing the heterogeneity of a causal effect (i.e., here firm size), such that part of the full effect can be identified. Previous studies have either used instrumental variables approaches or matching estimators [8]; however, these methods are highly problematic in our setting. First, both matching estimators and Heckman estimators in principle solve the selection problem inherent in estimating the effects of self-employment. However, for both types of estimators, the degree to which the problem is actually solved rests on how precisely exogenous and *observable* factors can identify the selection process. If this process is only weakly identified, Heckman estimators yield highly unstable and unreliable estimates [63], while the results of matching estimators approach those of simpler OLS results and are thus equally subject to selection bias. We argue that this problem is highly to be of concern in our context, as the selection process is mostly affected by personality traits and other factors that are essentially unobserved, and known selection factors such as preferences for autonomy and institutional features are also known determinants of well-being for most other people. In addition, even when they function well, neither Heckman procedures nor matching estimators solve the problem of survival bias, as some share of individuals who enter self-employment / entrepreneurship fail and thus also exit. This problem creates survival bias, which these methods are unlikely to alleviate.

Second, contrary to many other studies, we include data from 32 different countries in which the selection into entrepreneurship arguably varies with both personal characteristics and institutional factors that affect entrepreneurial incentives [1]. This makes it even less likely to find observed factors enabling us to match self-employed with similar respondents, as the proper matching procedure may vary from country to country and depend on institutional factors that themselves affect well-being directly. Similar problems apply to the use of instrumental variables approaches.

The method outlined in [17] has been used as an alternative to matching and instrumental variables by, for example, [18, 19] in the challenging context of establishing causality in the literature on the effects of foreign aid. The method rests on the fact that if a causal effect is known or credibly hypothesized to vary with some exogenous characteristic, the endogeneity bias in the *average* estimate is likely to be independent of the variation from the exogenous characteristic. This means that the heterogeneity, implemented as an interaction between a potentially endogenous and an exogenous variable, can be interpreted as a causal although perhaps partial effect. Determining causality from an interaction means that the implied *causal* effect we identify is a lower bound on the true effect, which depending on the severity of the selection problem can be anywhere between the lower bound and the full, un-interacted estimate: With severe selection bias, only the interaction difference is likely to reflect an actual causal effect, while with no selection bias, the full average estimate reflects a true causal influence.

We implement the method in [17] with the combination of self-employment and establishment size for the following reason: When we observe respondents who are self-employed, they have all self-selected into this type of employment at some point in time. The personal characteristics and experiences that create the problematic selection bias are therefore approximately the same for all self-employed respondents. However, when establishment size exceeds some limit, self-employed individuals typically undertake the original entrepreneurial tasks to a much smaller extent, if at all. Their span of control within the hierarchy has typically increased dramatically, and they are in general considerably more taken up with less diverse, repetitive, administrative tasks than with novel, entrepreneurial tasks. Note that the validity of the approach still requires that firm is independent of the error term. We have no way of testing for this assumption, but observe that firm size *per se* is not robustly associated with subjective well-being. In addition, present firm size is arguably exogenous to the choice of entering entrepreneurship, which was taken sometime in the past when the current firm was unlikely to exist. As such, firm size is in all cases exogenous in the sense of being pre-determined, and therefore *apriori* valid.

They therefore enjoy the autonomy and engage in the kind of tasks that we hypothesize affects their well-being to a correspondingly smaller extent. As such, the only crucial assumption that is necessary for clean identification in our case is that characteristics such as personality traits and intelligence do not change much over time. This assumption seems confirmed by most studies [43]. Following [17], any *additional* well-being effect that is enjoyed by being self-employed in small establishments can therefore be interpreted directly causally. In addition, this difference may be biased downwards because the risk of failing and thus the survival bias inherent in the estimate of self-employment—that is, we observe only those who either succeed or have not yet succumbed to competitive pressure or for other reasons left self-employment—is much more severe when we observe the association with well-being for those individuals self-employed in larger establishments. As noted above, our central causal estimates are therefore likely to be conservative.

Similarly, in further tests we effectively introduce a triple-interaction between self-employment, establishment size and a dummy capturing whether the respondent has been unemployed at any time during the last five years. We do so to correct for a separate cause of selection bias due to different motives for becoming self-employed: Entering self-employment from a state of unemployment is likely to be evidence of necessity entrepreneurship, which should not offer the same well-being dividend as opportunity entrepreneurship. We thereby move closer to establishing a true estimate of the causal influence of entrepreneurship, although including second-order interactions is also likely to yield a conservative estimate of the effect.

## Results

### Entrepreneurship and happiness

We start by observing that individuals who are self-employed are on average about .18 points more satisfied with their lives, compared to other citizens in their country with other employment. This lends initial support to Hypothesis 1, and is in conformity with earlier findings in the literature. In Table 2, column 1, we estimate how much of this difference is due to personal characteristics and other observable individual factors.

**Table 2:** Main results.

	1	2	3	4
Sample	All	No immigrants	No first gen.	Only immigrants
Social trust	.128*** (.005)	.125*** (.005)	.128*** (.005)	.134*** (.012)
Political confidence	.075*** (.009)	.076*** (.009)	.075*** (.009)	.076*** (.017)
Institutional confidence	.126*** (.006)	.124*** (.006)	.124*** (.006)	.137*** (.009)
Religiosity	.039*** (.006)	.041*** (.007)	.041*** (.006)	.033*** (.008)
Left-right placement	.085*** (.007)	.084*** (.007)	.084*** (.007)	.096*** (.012)
No placement	-.031 (.033)	-.040 (.037)	-.031 (.036)	-.031 (.042)
Female	.032 (.022)	.035 (.023)	.034 (.023)	.017 (.044)
Age	-.066*** (.006)	-.066*** (.006)	-.066*** (.006)	-.056*** (.006)
Age squared	.001*** (.000)	.001*** (.000)	.001*** (.000)	.001*** (.000)
Living with partner	.145*** (.018)	.159*** (.018)	.149*** (.019)	.093*** (.031)
Children at home	.074** (.021)	.078** (.023)	.079** (.023)	.022 (.039)
Children moved out	.155*** (.027)	.158*** (.027)	.155*** (.027)	.172** (.076)
Income decile	.100*** (.009)	.098*** (.009)	.099*** (.009)	.115*** (.015)
First generation immigrant	-.125** (.050)			
Half immigrant	-.089*** (.022)		-.104*** (.022)	-.099 (.063)
Second generation immigrant	-.011 (.057)		.032 (.065)	-.033 (.127)
Retired	.054 (.049)	.057 (.054)	.045 (.053)	.186** (.077)
Unemployed	-.902*** (.069)	-.912*** (.079)	-.917*** (.077)	-.779*** (.065)
Agriculture plus	-.207*** (.049)	-.199*** (.053)	-.204*** (.051)	-.223** (.105)
Food and hotel services	-.093*** (.027)	-.082*** (.029)	-.088*** (.028)	-.132** (.064)
Self-employed	.152*** (.027)	.139*** (.029)	.144*** (.029)	.229*** (.052)
Year fixed effects	Yes	Yes	Yes	Yes
Country fixed effects	Yes	Yes	Yes	Yes
Observations	228,213	189,780	208,055	20,158
Countries	32	32	32	32
Within R squared	.136	.133	.134	.131
F statistic	341.68	332.79	386.11	433.84
*Separating types*				
Self-employed, below 25 employees	.259*** (.059)	.247*** (.069)	.251*** (.062)	.353** (.164)
Self-employed, below 100 employees	.144*** (.027)	.132*** (.031)	.136*** (.029)	.215*** (.059)
Self-employed, larger	.142*** (.042)	.099* (.055)	.125*** (.045)	.256 (.159)
Within R squared	.136	.133	.134	.131
F statistic	334.49	666.97	536.69	436.92

Note

*** (**) [*] denote significance at p < .01 (p < .05) [p < .10].

Starting with the control variables, we find that their signs conform to standard findings in the well-being literature [16]. Well-being is positively associated with social trust and institutional confidence, and somewhat weaker with political confidence. Richer, religious, and politically right-wing respondents who live with their partner also declare themselves more satisfied, as do people whose children have moved out, relative to those with children living at home. We also observe that the least satisfied tend to be the middle-aged. Finally, individuals who are unemployed, first-generation immigrants or who work in low-status jobs in agriculture, forestry, fishery, mining or hotel and beverage services also tend to be significantly less satisfied with their lives.

However, our main estimate suggests that self-employed individuals are approximately .15 points more satisfied with their lives. This well-being effect is robust to either excluding all immigrants (in column 2) or all first-generation immigrants (in column 3) and is precisely estimated. Yet, when restricting the sample to the roughly 20,000 immigrants in the European Social Survey, the point estimate increases substantially to .23, which can be construed as evidence in favour of Hypothesis 2. We thus find indications that the difference may be larger for immigrants, although part or all of this difference may be due to selection effects when people with a particular preference for risk-taking or self-employment migrate. As such, the self-employed migrants may be particularly pertinent examples of the general methodological problem of self-selection into entrepreneurship.

### Selection and the importance of firm size and voluntary choice for well-being

In the lower panel of Table 2, we employ our version of the identification method in [17]. We observe throughout, consistently with Hypothesis 4, that the difference between the well-being estimate of entrepreneurship in small and medium-sized establishments in the self-employment estimate is approximately .12 points. Although not shown here, we note that the difference is robust to excluding single countries from the sample. It is therefore unlikely to be driven by well-known complications such as the very high self-employment levels for Italy and Greece. Excluding these cases, the difference that can be causally interpreted tends to increase slightly. We also find no robust well-being differences between individuals in regular employment in small versus medium-sized establishments, indicating that the differences mainly apply to the self-employed). As we noted above, the difference between the well-being estimate of entrepreneurship in small and medium-sized establishments in the self-employment estimate can be interpreted causally. Part of the remaining difference between self-employed individuals and those employed otherwise is .14, which cannot be interpreted directly as it results as a mix of potential causal influence, selection effects and unobserved survival bias.

In Table 3, we further explore the selection problem in two different ways. In columns 1 and 2, we separate immigrants from non-Western and Western countries, identified as those above a real GDP per capita of USD 15,000. In column 3, we exclude individuals working in agriculture, forestry, fishery and mining, and in column 4, we further exclude individuals in hotel and beverage service. The reason for both types of separation of the data is that we exclude groups that are more likely to enter self-employment out of necessity—non-Western immigrants and individuals in traditional sectors—from those more likely to enter self-employment as an opportunity.

**Table 3:** Specific results.

	1	2	3	4
Sample	Non-Western imm.	Western imm.	Not agric.	Not agri. hotel
First generation immigrant			-.129*** (.049)	-.131*** (.047)
Half immigrant	-.155 (.192)	-.096 (.125)	-.087*** (.023)	-.086*** (.024)
Second generation immigrant	.156 (.349)	.000 (.229)	-.008 (.058)	-.006 (.057)
Agriculture plus	-.207 (.253)	-.176 (.146)		
Food and hotel services	-.231* (.124)	.061 (.096)	-.094*** (.027)	
Self-employed	.019 (.114)	.333*** (.088)	.141*** (.026)	.148*** (.027)
Year fixed effects	Yes	Yes	Yes	Yes
Country fixed effects	Yes	Yes	Yes	Yes
Observations	4847	8018	215,846	204,646
Countries	30	32	32	32
Within R squared	.142	.137	.135	.135
F statistic	3333.15	1037.47	312.81	400.66
*Separating types*				
Self-employed, below 25 employees	.637** (.299)	.127 (.278)	.249*** (.062)	.245*** (.067)
Self-employed, below 100 employees	-.027 (.141)	.314*** (.099)	.131*** (.027)	.139*** (.027)
Self-employed, larger	-.089 (.331)	1.012*** (.324)	.141*** (.043)	.151*** (.044)
Within R squared	.142	.138	.155	.135
F statistic	-	977.53	297.50	398.20

Note

*** (**) [*] denote significance at p < .01 (p < .05) [p < .10]. The full specification is included, but not shown.

The results in Table 3, columns 1 and 2, must be interpreted with care as they are based on relatively small samples. In particular, the vast majority of self-employed immigrants from non-Western countries are engaged in small establishments, while the majority of Western immigrants are engaged in medium-sized industries. We find that only self-employment in small establishments, for the former group, and self-employment in medium-sized industries, for the latter group, is significant. For the latter group, in which we have a strong self-selection problem, we thus observe that success seems to matter. For the former, we find again find the strongest effects for the group least likely to self-select. Conversely, we find no evidence that self-employment in traditional industries in any way biases our overall findings although these industries are likely to attract more necessity entrepreneurship and hide actual unemployment. Again, these findings are robust to excluding single countries or years.

Finally, in Table 4 we apply what is equivalent to a second-order interaction between self-employment, establishment size and the dummy capturing if respondents have been unemployed during the last five years. As such, we separate respondents who probably enter self-employment as an opportunity from those likely to have entered self-employment out of necessity as a way out of unemployment, which provides an additional test of Hypothesis 2. Given that the true well-being effect of self-employment is due to undertaking entrepreneurial tasks of one’s own choosing or close to one’s own preferences, and not merely a reflection of self-selection or unobserved personality traits, we should observe that respondents in small establishments without a recent history of unemployment ought to report significantly higher levels of well-being.

**Table 4:** Separating opportunity and necessity.

	1	2	3	4
Sample	All	No immigrants	No first gen.	Not agri. hotel
Self-employed, below 25 employees	-.295 (.191)	-.220 (.177)	-.248 (.186)	-.289 (.188)
Self-employed, below 100 employees	.053 (.050)	.046 (.057)	.052 (.053)	.042 (.053)
Self-employed, larger	-.121 (.174)	-.010 (.162)	-.049 (.155)	-.275 (.196)
Not unemployed last 5 yrs	.204*** (.023)	.212*** (.029)	.196*** (.025)	.210*** (.021)
Not unempl * Self-employed, below 25	.647*** (.213)	.485** (.231)	.588*** (.223)	.625*** (.221)
Not unempl * Self-employed, below 100	.058 (.059)	.018 (.068)	.040 (.064)	.071 (.065)
Not unempl * Self-employed, larger	.474* (.261)	.238 (.232)	.345 (.222)	.662 (.306)
Year fixed effects	Yes	Yes	Yes	Yes
Country fixed effects	Yes	Yes	Yes	Yes
Observations	60,737	48,748	54,324	54381
Countries	32	32	32	32
Within R squared	.151	.152	.150	.149
F statistic	-	1541.32	9422.46	-

Note

*** (**) [*] denote significance at p < .01 (p < .05) [p < .10]. The full specification is included, but not shown.

As is evident in Table 4, this is the difference we observe for the equivalent of a second-order interaction. Our main identified effect, which we can interpret as credibly causal, is driven by self-employed respondents in smaller establishments who have not entered self-employment from a status of recent unemployment. Comparing these individuals to similar respondents without a recent unemployment history in larger establishments, our estimates suggest a precisely measured lower bound of the true causal effect of entrepreneurship on well-being of about .2 points. As is the case for previous estimates, these last findings are robust to excluding single countries or years.

### The importance of autonomy for well-being and self-employment

We thus identify significant and sizeable associations with well-being of voluntary opportunity self-employment in relatively small firms. We argue that these associations can be interpreted causally. However, while most of the small existing literature interprets the general finding as evidence of increased autonomy, which entrepreneurs may have a particular preference for, we note above that there are at least two other possible explanations. In Table 5, we therefore directly test the autonomy hypothesis (Hypothesis 3) by including actual perceived autonomy in column 1 and individuals’ stated preferences for autonomy in column 3. In columns 2 and 4, we test whether the specific types of self-employed respondents in the European Social Survey also in fact experience stronger autonomy and have stronger autonomy preferences than other respondents.

**Table 5:** The importance of autonomy.

	1	2	3	4
	Satisfaction	Autonomy, act.	Satisfaction	Autonomy, pref
Sample	All	All	All	All
Self-employed, below 25 employees	-.566*** (.192)	2.435*** (.239)	-.340* (.194)	-.429*** (.082)
Self-employed, below 100 employees	-.126* (.065)	3.009*** (.135)	.037 (.053)	-.291*** (.034)
Self-employed, larger	.016 (.213)	2.857*** (.358)	-.107 (.185)	-.245** (.109)
Not unemployed last 5 yrs	.174*** (.025)	.263*** (.044)	.209*** (.023)	.022* (.013)
Not unempl * Self-employed, below 25	.768*** (.224)	-.565*** (.341)	.626*** (.226)	.204 (.153)
Not unempl * Self-employed, below 100	.111 (.077)	-.462*** (.093)	.062 (.061)	-.044 (.034)
Not unempl * Self-employed, larger	.225 (.267)	-.155 (.408)	.357 (.258)	-.021 (.166)
Autonomy, actual	.055*** (.006)			
Autonomy, preference			-.063*** (.022)	
Year fixed effects	Yes	Yes	Yes	Yes
Country fixed effects	Yes	Yes	Yes	Yes
Observations	45,174	45,276	58,592	58,768
Countries	32	32	32	32
Within R squared	.147	.113	.151	.021
F statistic	-	-	-	-

Note

*** (**) [*] denote significance at p < .01 (p < .05) [p < .10]. The full specification from Table 2 is included, but not shown.

The first finding from the estimates, which include the same baseline specification as in previous tables although we do not show all results, is that both actual perceived autonomy and preferences for autonomy are associated with well-being. Controlling for perceived autonomy, the choice of self-employment from a previous state of unemployment—that is, a choice which we interpret as necessity entrepreneurship—is significantly negatively associated with well-being. However, when comparing the specific heterogeneous effects of self-employment for those engaging in opportunity entrepreneurship in small and medium-sized firms in Table 4 with those in Table 5, it appears that adding autonomy does *not* change the estimates. In other words, the positive effects on well-being of being self-employed cannot be driven by perceived autonomy (column 1) or preferences for autonomy (column 3).

In addition, we observe that the self-employed express stronger preferences for autonomy than those who are either unemployed or in ordinary employment, and that those in small firms have stronger preferences than self-employed individuals in medium-sized or larger firms. However, we do not observe any differences between individuals who likely entered self-employment from unemployment—that is, those more likely to have chosen self-employment out of necessity—and individuals who entered from employment. As such, the pattern of preferences for autonomy and perceived autonomy does not match the pattern of well-being effects of self-employment. Since the inclusion of autonomy among the determinants of well-being also does not change the estimates, we find no compelling evidence in support of the main explanation of the association between entrepreneurship and well-being offered in the existing literature. In other words, the main well-being effects of voluntary self-employment must be driven by some other mechanism than autonomy. The sixth wave of the European Social Survey includes more questions about job conditions etc. that may be used to assess alternative theoretical mechanisms. However, given our causal approach, the existence of these questions in only one wave of the survey means that we are slicing the data very thinly when trying to test separate mechanisms. As a consequence, the results proved to be unstable and we therefore refrain from using this otherwise interesting option in the survey. We turn to discussing these potential mechanism in the final section.

## Concluding discussion

### Contribution to the literature

Our first contribution is to replicate existing findings concerning entrepreneurship and well-being, but we do this by means of a novel approach that gets closer to causal identification than in earlier contributions. Thus, we find substantial evidence across a large individual-level sample of more than 200,000 respondents in 32 European countries that the well-being effect of self-employment is causal and generalizes to most Western countries. Using a novel method to identify causal effects, which does not require any knowledge or assumptions about the selection process into entrepreneurship, we also show that at least a substantial part of the known association can be interpreted as evidence of a causal influence of what appears to be voluntary, that is, opportunity-motivated, self-employment. The conditions under which this causal effect occurs is consistent with situations in which self-employed individuals are more likely to experience more autonomy, exercise more judgement, have more creative and entrepreneurial tasks, and experience more task diversity. However, when directly testing the most prevalent theory of how entrepreneurship leads to more well-being—that entrepreneurs may both prefer and experience substantially more autonomy and are more satisfied when being self-employed—we find no evidence in favour of the theory. It may therefore be necessary to consider other transmission mechanisms such as task-specific preferences or entrepreneurial preferences for diversity and frequent situations that require individual judgement.

Our contribution to the existing literature is thus a *combined* approach to filling two substantial gaps in the literature in addition to a novel finding that runs counter to the present understanding of well-being effects of entrepreneurship.

First, studies that have solved or alleviated problems associated with self-selection and survival bias have tended to achieve this within limited samples of only one or a few countries [6, 8]. The findings of these studies may thereby not generalize to most Western countries where historical and institutional differences tend to create very different environments for entrepreneurial activity. Second, studies using larger country samples that are able to yield generalizable results have not been able to credibly solve the inherent endogeneity problem arising out of self-selection and survival. Employing the approach recently developed in [17] to deal with causal inference in challenging empirical settings, we alleviate both problems and provide conservative estimates of a true treatment effect of the voluntary choice of entering self-employment and entrepreneurial activity. Finally, we find evidence that cannot be consistent with the prevalent explanation of the well-being effects of entrepreneurial activity, as the direct inclusion of a measure of perceived autonomy does not change the basic estimates and the pattern of associations between self-employment and autonomy far from matches the pattern between self-employment and well-being.

We observe a simple average difference between self-employed individuals and everyone else in their country of approximately .18 points and show that we can credibly identify an average treatment effect of self-employment of approximately .12 points. As such, we show that the potential methodological bias in simple comparisons with insufficient control of individual-level factors is about 50%, but that the *treatment effect* of going into self-employment and undertake some form of entrepreneurial activity is equivalent to an income increase of slightly more than moving one decile up. However, we also emphasize that despite its non-negligible size, this estimate is a lower bound of a “true” treatment effect. We moreover find evidence that is consistent with a crucial influence of the type of entrepreneurial motivation: Separating self-employment out of necessity from opportunity-motivated self-employment through information on respondents’ unemployment history, we find that while the effect of the former may be zero, the lower-bound effect of what may be taken as voluntary, opportunity-motivated self-employment is about .2 points.

One way of interpreting our findings is that the well-being effect of entering self-employment mainly derives from the active choice and engagement in entrepreneurial activity. This is consistent with findings in [29], which finds that the well-being effect of self-employment arises primarily for particularly growth-aspiring entrepreneurs not encumbered by poor institutions or policies. This is indeed consistent with further patterns in the data emerging when we separate our sample according to whether countries have small or large government burdens, measured by the Heritage Foundation [64] indices. Both the average well-being estimate as well as the difference between self-employment in small and medium-size establishments (i.e. our true causal estimate) are significantly larger in countries with small government sectors, consistent with the findings in [65]. Our findings are also consistent with the focus in [43] on the importance of self-determination and indeed lends credence to the value of entering into self-determining, entrepreneurial *activities* as opposed to merely having the *opportunity*.

However, the procedural aspects of self-employment may perhaps be insufficient for the choice of self-employment to have any effect on individual, subjective well-being. The bulk of the effect, which we can establish as causal, is associated with the choice of being self-employed in small enterprises in which it is mostly unavoidable that the owner / entrepreneur engages in directly entrepreneurial tasks. These tasks, which separate entrepreneurs from manager-owners, appear directly associated with well-being. In combination with the finding that autonomy cannot be the cause of the effects, we believe it is fair to hypothesize that the effects arise due to the entrepreneurial tasks that are specific to small, young firms and potentially also the diversity of tasks that the self-employed perform in such firms.

### Future research

While we report substantial evidence in favour of not only a causal effect of self-employment, but also direct effects of engaging in entrepreneurial tasks, our choice of survey is insufficient to establish that this is also the way entrepreneurs and otherwise self-employed respondents subjectively perceive their individual situation. In this research, we interpret the well-being effects not only as procedural utility and consequences of having opportunity, but as evidence of the importance of entrepreneurial tasks and activities. Yet, without specialized surveys or long-run experimental designs, it remains an open question how to interpret the causal effects of choosing a life as self-employed over any other alternatives that an individual has.

Relatedly, while our findings suggest that there is a “size effect” when it comes to entrepreneurs’ subjective well-being, we cannot disentangle the reasons for such an effect. We have suggested that the decline in subjective well-being that is associated with a larger firm size may depend on the transformation of the founder-entrepreneur’s role towards a more traditional management role as the venture grows. This implies engaging more in predictable, routine tasks that absorb a larger amount of the entrepreneur’s time and means correspondingly less time for engaging in more autonomous or diverse entrepreneurial tasks. In terms of self-determination theory b this may be interpreted as a change from autonomous towards controlled motivation that may lead to less reported well-being. However, while we can reject simple autonomy explanations, we unfortunately cannot directly measure such mechanisms. Similar remarks apply to our finding that there seems to be an industry effect when it comes to entrepreneurs’ reported well-being. Our findings are consistent with a paramount influence of self-determination and opportunity choice on well-being, but no direct evidence of which type or which specific tasks. We therefore conclude this paper by noting that our paper is the first to simultaneously tackle the causality problem and the generalizability of previous findings. As such, we provide evidence of a causal effect while the particular motivation for why the effect occurs must remain a puzzle for now.
